# Canadian Stroke Best Practice Guidance During the COVID-19 Pandemic

**DOI:** 10.1017/cjn.2020.74

**Published:** 2020-04-17

**Authors:** Eric E. Smith, Anita Mountain, Michael D. Hill, Theodore H. Wein, Dylan Blacquiere, Leanne K. Casaubon, Elizabeth Linkewich, Norine Foley, Gord Gubitz, Anne Simard, M. Patrice Lindsay

**Affiliations:** Department of Clinical Neurosciences, Cumming School of Medicine, University of Calgary, Calgary, Alberta, Canada; Divisions Physical Medicine and Rehabilitation, Department of Medicine, Dalhousie University, Halifax, Nova Scotia, Canada; Department of Neurology and Neurosurgery, McGill University, Montréal, Quebec, Canada; Division of Neurology, Department of Medicine, University of Toronto, Toronto, Ontario, Canada; Toronto Western Hospital Stroke Program, University Health Network, Toronto, Ontario, Canada; Division of Neurology, Faculty of Medicine, University of Ottawa, Ottawa, Ontario, Canada; WorkHORSE Consulting Inc., London, Ontario, Canada; Sunnybrook Health Sciences Centre, Regional Stroke Centre, Toronto, Ontario, Canada; Department of Occupational Science and Occupational Therapy, University of Toronto, Toronto, Ontario, Canada; Department of Medicine (Neurology), Dalhousie University, Halifax, Nova Scotia, Canada; Heart and Stroke Foundation of Canada, Toronto, Ontario, Canada

**Keywords:** Stroke, Coronavirus 2019, COVID-19, Clinical practice guidelines, Telestroke, Rehabilitation, Prevention, Knowledge translation

## Stroke Best Practice Guidance During the COVID-19 Pandemic

Guidance from the Heart and Stroke Foundation of Canada Canadian Stroke Best Practices Advisory Council.

*The French translation of this manuscript is available as Appendix 2 in the online-only supplementary material.*

## Introduction

The worldwide pandemic of coronavirus disease 19 (COVID-19) caused by the severe acute respiratory syndrome coronavirus 2 (SARS-Co2) has emerged as one of the biggest public health crises in a century. Health systems in Canada face immense challenges, both to cope with the number of affected patients and the constraints imposed by containment measures such as physical distancing, quarantine, and personal protection. Stroke care across the globe and within Canada is rapidly changing to meet these challenges^1-7^ (see Appendix 1 in Supplementary material).

This document provides guidance on implementing evidence-based stroke care during the COVID-19 pandemic, based on expert opinion from the Canadian Stroke Best Practices Advisory Council. Despite incomplete and rapidly evolving evidence, we offer early guidance without formal recommendations and evidence grades because urgent changes are necessary. This guidance is based on expert opinion and early shared experiences with reorganizing stroke systems at the time of writing (April 13, 2020).

This document is guided by two main principles. First, stroke remains a medical emergency and should be treated as such. Second, stroke care is highly effective. Stroke best practice recommendations remain as evidence-based and relevant as ever, even though logistics and workflows need to change to accommodate the pandemic. Evidence-based stroke care reduces mortality, length of stay, improves functional outcomes, and prevents recurrence, contributing to relief of the health system.^8-10^ It should not be intentionally stopped or suspended, and this is universally agreed upon by stroke leaders (Appendix 1 in Supplementary material).

Across the globe, stroke centers are reporting decreased number of individuals with stroke symptoms presenting to emergency departments for care, especially those with TIA or milder symptoms. The causes behind this are not clear at this time. There is anecdotal evidence that patterns of patient engagement with health care services may be changing during the pandemic. Such decreases may raise new concerns, as individuals reluctant to seek medical care may be at a higher risk of a recurrent event with more severe and lasting physical, cognitive, and emotional impacts without timely assessment and treatment.

## Stroke Awareness Recognition and Response

Stroke is a medical emergency. This fact is not altered by the COVID-19 pandemic. Public awareness campaigns and existing processes in place for emergency medical system response to stroke should be maintained. Active public awareness efforts are needed to reinforce this message and reduce delays in seeking medical assistance.

## Hyperacute Stroke Care

Acute stroke activations pose a risk because the stroke team must come in close contact with patients from the community, many of whom will have uncertain COVID-19 status. Patients unable to answer COVID-19 screening questions due to aphasia, cognitive issues, or encephalopathy should be treated as suspected COVID-19 positive. Personal protective equipment (PPE) should be worn, in accordance with local policies. Guidance for a “protected code stroke pathway” has been offered, emphasizing screening, appropriate use of PPE, where each team member understands their specific role, to minimize potential COVID-19 exposure.^1,4^ Telemedicine can be used for acute stroke consultation to avoid exposing team members and reduce use of PPE.

Endovascular thrombectomy (EVT) is a highly effective stroke treatment indicated for severely affected ischemic stroke patients at risk for respiratory instability, vomiting, aspiration, and coughing, all of which could increase spread of viral-laden droplets. To avoid the risk of emergency intubation in the EVT angiography suite with potential viral spread, it is appropriate to make early decisions regarding the need for intubation. If needed, it should be done in an elective, controlled manner prior to transfer to the angiography suite in a negative pressure room. This does not imply the need to intubate more patients, and we continue to recommend that monitored anaesthesia is preferred unless there is a clinical indication for intubation.^5^ Similarly, recent multisociety guidance agrees that intubation is not necessary for all suspected or confirmed COVID-19 patients.^11,12^ The minimum possible sedation should be used in COVID-19 suspected patients, to reduce the risk that bag-mask ventilation, an aerosol-generating procedure, would be required.^11^

Demand for intensive care unit (ICU) beds may exceed supply during the surge of COVID-19 patients. It is appropriate to consider predicted stroke-related mortality as one of the criteria for ICU admission. However, many patients with stroke, including hemorrhagic stroke, can be saved with intensive care and will have a lower expected mortality than similar-aged COVID-19 patients with acute respiratory distress syndrome. As recommended by ethicists, decisions on triage should be based on objective evidence for mortality risk without making presumptions about quality of life for stroke survivors.^13^ Triage for ICU admission should be made only after attempting emergency procedures to reverse the patient’s condition, including intravenous thrombolysis and EVT for acute ischemic stroke and treatment of hydrocephalus in hemorrhagic stroke patients.

We encourage clinicians to amend their practices but not to deviate from evidence-based care.^5^ We do not suggest amending acute stroke computed tomography (CT)-angiography protocols to include CT of the chest to look for signs of COVID-19. The diagnostic value of CT chest for COVID-19, including false positive and negative rates, is not well defined at present. We also do not suggest substituting tenecteplase for alteplase for thrombolysis even though tenecteplase is more convenient to infuse, requiring shorter duration of contact with the patient. There is insufficient evidence that tenecteplase is equivalent to alteplase, and the optimal dose is not clear.

Key Messages

1. Stroke is a medical emergency irrespective of the pandemic and existing evidence-based stroke guidelines should continue to be followed.
2. There is a need to continue to raise awareness with the public that stroke is a medical emergency, and they need to seek medical attention without delay despite COVID concerns.
3. Hyperacute stroke response teams remain available to treat acute stroke.
4. Changes in workflow processes are required within a Protected Code Stroke model.
5. Intubation is not necessary for all suspected or confirmed COVID-19 patients undergoing EVT.

## Inpatient and Stroke Unit Care

Stroke unit care, defined as care by an experienced interdisciplinary team with colocation of patients on a designated inpatient unit, reduces disability and saves lives. Challenges to providing stroke unit care during the pandemic include reduced staff due to illness and redeployment and potential admission of COVID-19-positive stroke patients to general medical wards rather than stroke units.

There is already an increasing strain on health care resources in hospitals, including redeployment of highly skilled health care providers (HCP). As a result, stroke units may be staffed by nonstroke experts. Accordingly, hospitals should develop strong, team-based approaches to optimize best practice care for stroke patients using all team members’ capabilities. Patients with a dual diagnosis of acute stroke and COVID-19 may be admitted to a nonstroke unit with HCP less experienced at providing stroke care and early rehabilitation.^5^ In these instances, there should be processes in place for consultation with stroke experts and education on stroke best practices.^14^ Particularly important is education for the recognition, assessment, and management of dysphagia, aphasia, cognitive impairment, handling and positioning of hemiplegic extremities, venous thromboembolism prevention, transfers, and fall prevention. All interdisciplinary staff should also receive basic stroke education on assessment for signs and symptoms of stroke as part of monitoring for possible stroke transformation. This should include screening tools such as FAST (Face, Arms, Speech, Time)^15^ and protocols for in-hospital actions to be taken if signs and symptoms of stroke are identified. Additional education and support may be required for HCP caring for patients with intracerebral hemorrhage (ICH) and subarachnoid hemorrhage (SAH).

After patients receive hyperacute reperfusion treatment (thrombolysis and/or EVT), care is optimally provided in an intensively monitored unit or critical care bed. Where access to critical care beds becomes limited, this care could be provided in a ward bed with appropriate supports. Broadly speaking, this would include measures for enhanced patient monitoring particularly within the first 24 h post-hyperacute treatment, education of the interdisciplinary team regarding all aspects of care for thrombolysis and EVT patients, and clear communication between team members regarding patient clinical status. Patients should be cared for in an area with high visibility from the hall and ideally with cardiac telemetry.

Key Messages

1. Stroke patients should continue to be cared for in specialized acute stroke units where possible.
2. Education and basic skills training may be required for nonstroke experts caring for stroke patients to ensure patient safety and optimizing recovery.
3. Where access to critical care beds becomes limited, this care could be provided in a ward bed with appropriate supports.

## Stroke Rehabilitation

Access to rehabilitation care has been significantly reduced during the COVID-19 pandemic.^16,17^ People with stroke discharged directly to the community from acute care may have limited access to specialized stroke rehabilitation. Those who receive inpatient stroke rehabilitation may have a reduced length of stay.^1,18^ It remains vital that persons with stoke continue to have access to specialized inpatient, outpatient, early supported discharge, and community stroke rehabilitation. Stroke rehabilitation is essential for people to achieve an optimal physical, cognitive, emotional, communicative, and social functional level following stroke, as well as to prevent or slow future functional decline and secondary health conditions.^19^

Rehabilitation teams need to continue to follow evidence-based care for stroke patients.^19^ Rehabilitation teams should be well educated on the use of PPE with strict adherence to infection control procedures for direct contact therapies, shared equipment, and spaces to ensure safe access is maintained.^17^ Essential components of stroke rehabilitation care should be adapted to follow public health recommendations on physical distancing with consideration of such things as virtual team conferences.

Telerehabilitation is an effective and well-accepted method of providing outpatient and community rehabilitation services and is of particular importance during the COVID-19 pandemic.^20-22^ To support discharge planning, the use of telerehabilitation should be considered for family conferences, family and caregiver education and skills training, assessment of home environment, patient monitoring, and outpatient therapies. If telerehabilitation is planned for outpatient therapy, then education, skills training, and setup of the selected telerehabilitation platform for patients and family and caregivers residing with them should be provided prior to discharge. Patients should also be provided with clear discharge recommendations^19^ and instructions for continued rehabilitation at home. Consideration ought to be given for earlier follow-up for patients whose lengths of stays are shortened by COVID-19 policies to allow earlier identification of potential complications or functional decline. Telemedicine can also help identify those individuals whose changing needs and health status require an in-person assessment.^23^

People living in the community with chronic stroke will continue to require access to rehabilitation services. Rehabilitation professionals should ensure they have processes in place to triage referrals and address their needs to help prevent functional decline and complications during this time. Outpatient botulinum toxin injections during the COVID-19 pandemic should be considered when proper PPE is available and the individual is experiencing or is at risk of experiencing significant discomfort or pain, functional decline, or increased caregiver burden.

Key Messages

1. It is vital that persons with stoke continue to have access to specialized inpatient, outpatient, early supported discharge, and community stroke rehabilitation.
2. Essential components of stroke rehabilitation care should be adapted to follow public health recommendations on physical distancing and ensuring personal protection for staff and patient when direct contact is required.
3. Telerehabilitation is an effective and well-accepted method of providing outpatient and community rehabilitation services and is of particular importance during the COVID-19 pandemic.

## Secondary Prevention of Stroke Care

During the pandemic, access to specialized secondary stroke prevention services^24^ may be limited. In-person outpatient assessments have been strongly discouraged in many health jurisdictions. Most stroke preventive care during the pandemic will need to be delivered by telemedicine, and evaluations should be modeled along the topics defined in the Post Stroke Checklist and core elements of stroke prevention care.^25,26^ Lifestyle management issues of secondary prevention such as diet, exercise, weight, alcohol intake, and smoking should be addressed and may be impacted by public health policies that recommend staying at home. Patients, families, and caregivers should be provided with education, strategies, and resources for self-management.^27^ It is possible to complete some elements of a neurological exam via telemedicine with direct exam (e.g., mental status and speech), observation (e.g., portions of cranial nerve exam, extremity motor exam, coordination and gait), or with assistance of another person accompanying the patient (sensory exam).^28^ This is essential when evaluating for occurrence of a new event. Patients should be asked whether they have a home blood pressure unit and glucometer (if applicable) for ongoing monitoring of secondary prevention targets.

For cases requiring in-person care, rapid assessment TIA clinics and stroke prevention clinics will need to have mechanisms in place to screen all patients for COVID-19 symptoms prior to arrival, and appropriate PPE measures and equipment should be available in these clinics. Individuals presenting within 24 hours should continue to have urgent brain and vascular imaging (e.g. CT/CTS scans) and electrocardiogram performed. It is advised that health professionals requesting urgent neuro (vascular) imaging communicate directly with a radiologist to ensure that the imaging can be completed in a timely manner as normal request workflows may be interrupted. Other investigations should be completed as soon as possible according to guidelines, acknowledging that some diagnostic services (e.g., echocardiography) may not be available during the pandemic. Individuals outside of the 24-h timeframe should have investigations completed as defined in the stroke best practice algorithm.^24^ Direct admission is suggested for cases requiring hospitalization to reduce ED burden.

Key Messages

1. Secondary prevention services and follow-up must continue to be implemented to reduce recurrent stroke incidence, with revised workflows.
2. Telemedicine-enabled evaluation should be modeled on the topics defined in the Post Stroke Checklist and core elements of stroke prevention care.^25,^ ^26^
3. Individuals presenting within 24 hours should continue to have urgent brain and vascular imaging (e.g. CT/CTA scans) and electrocardiogram performed.

## Telestroke Across the Continuum

During the COVID-19 pandemic, telemedicine has been rapidly adopted by many health systems to facilitate care provision while maintaining physical distancing and reducing the risk of nosocomial viral transmission. Telestroke systems for hyperacute stroke care and support in decision-making for thrombolysis and EVT care are well established.^20^ Telemedicine can provide remote access to stroke specialists, sparing the need for transfer to tertiary care centers. It preserves the stroke specialist workforce by avoiding the risk of in-person exposure and infection and reduces use of PPE.^1^ In institutions without current telestroke systems, other telemedicine supports using videoconferencing software, shared imaging access, and telephone consultation may be implemented to assist in hyperacute stroke decision-making.^29,30^

Toolkits based on current evidence and expert opinion are available within the Canadian Stroke Best Practice Recommendations (CSBPR) to help inform services that are switching to virtual care within short timelines.^20,31^

Stroke care providers and health systems should be aware of potential barriers to access such as patients and caregivers without reliable internet services or access to devices. Barriers to use of technology in individuals with stroke and communication, cognitive, or physical impairments should also be considered. Other social determinants of health such as stable housing may also create challenges in using virtual modalities to receive health care services. Telephone visits or involvement of family members in the assessment process could address these barriers.

Key Messages

1. Telestroke systems for hyperacute stroke care and support in decision-making for thrombolysis and EVT care are well established, and implementation should be expanded to service all regions.
2. Toolkits based on current evidence and expert opinion are available within the CSBPR to help inform services that are switching to virtual care within short timelines.
3. Barriers to access and utilization should be considered and work-around solutions implemented.

## Summary

The COVID-19 pandemic has drastically changed the processes of patient access and stroke care. Yet, the nature and quality of stroke care across the whole continuum has proven benefits on long-term outcomes. Standards and comprehensiveness of care for stroke patients must be preserved; otherwise, the rate of recurrent stroke and ongoing functional, cognitive, and social disabilities will rise and create a new burden on an already over-stressed system. Overall, measures to sustain best practice stroke care should be implemented within pandemic planning across the health system. Alternate care models can allow continued access to stroke care for those who need it throughout and beyond the COVID-19 pandemic.

## Supplementary material

For supplementary material accompanying this paper visit http://dx.doi.org/10.1017/cjn.2020.74. click here to view supplementary material
