# Supplementary material for: Canadian Stroke Best Practice Guidance During the COVID-19 Pandemic
Source: Can J Neurol Sci. 2020 Apr 17:1–5. doi: 10.1017/cjn.2020.74 (PMC7235308; doi:10.1017/cjn.2020.74)
Supplement: Supplementary file 1 [file S0317167120000748sup.zip › S0317167120000748sup001.docx]

**Title:** **Canadian Stroke Best Practice Guidance During the COVID-19 Pandemic**

**Supplemental Material: Summary of Recent Statements on Stroke Care during the COVID Pandemic**

**Authors:**

Eric E. Smith, MD, PH, Anita Mountain MD, BScOT; Michael D Hill MD, MSc; Theodore H. Wein MD; Dylan Blaquiere MD; Leanne K. Casaubon MD, MSc; Elizabeth Linkewich TO (ON Reg), MPA; Norine Foley RD, MSc; Gord Gubitz MD, Anne Simard B Journ, MSc; Patrice Lindsay RN, PhD, *on behalf of the Canadian Stroke Best Practices Advisory Council*.

**Corresponding Author:**

M. Patrice Lindsay, RN, PhD

Director, Systems Change and Stroke Program

Senior Editor, Canadian Stroke Best Practice Recommendations

Heart and Stroke Foundation of Canada

2300 Yonge Street, Suite 1300, P.O. Box 2414

Toronto, Ontario, Canada M4P 1E4

Ph: +1-647-943-3042

[patrice.lindsay@heartandstroke.ca](mailto:patrice.lindsay@heartandstroke.ca)

**Funding**

The development of the CSBPR is funded in its entirety by the Heart and Stroke Foundation of Canada. No funds for the development of these guidelines are received from commercial interests, including pharmaceutical and medical device companies.

**Stroke Best Practice Guidance During the COVID-19 Pandemic**

Guidance from the Heart and Stroke Foundation of Canada Canadian Stroke Best Practices Advisory Council

**Appendix 1: Global scan of stroke care statements and guidance during COVID-19**

| Guideline | Topic | Recommendations  (Abbreviated in some cases – refer to source document for full details) |
| --- | --- | --- |
| Lyden P.  Temporary Emergency Guidance to US Stroke Centers During the COVID-19 Pandemic.  *Stroke*;0(0) doi: doi:10.1161/STROKEAHA.120.030023 | **Guideline Adherence** | Continue treating stroke patients as appropriate. Full adherence to guidelines may be challenging but needed treatment should be offered to the extent possible. |
|  | **Personal protective equipment (PPE)** | Seek ways to minimize the use of scarce PPE in your medical center. Send fewest possible team members to see Code Stroke patients, and into rooms for follow up visits. |
|  | **Telemedicine** | Telemedicine began with telestroke. The NIHSS can be performed efficiently via telemedicine.  Televideo is superior to telephone. Yet telephonic consultation is superior to no consult. |
|  | **Health and Safety** | Health and Safety. Take care of yourselves, your families, and your teammates. Follow protocol (local, CDC and WHO), including guidelines for hand washing, PPE use, COVID-19 testing and evaluation, and self-quarantine as needed. |
|  | **Teamwork** | Teamwork. Stroke care has always been a multispecialty, collaborative effort among EMS, physicians and nurses from the Emergency Departments to the Stroke Units, ICUs and Rehabilitation Centers.  Collaboration, collegiality, and compassion for one another are crucial to making it through this challenge. A true sense of a unified Stroke System of Care is needed now more than ever. |
| NHS (Macchine D)  Clinical guide for the management of stroke patients during the coronavirus pandemic  23 March 2020; Version 1(abbreviated)  <https://www.england.nhs.uk/coronavirus/wp-content/uploads/sites/52/2020/03/C033-Specialty-guide_-Stroke-and-coronavirus-v1-24March_.pdf> | **Hyperacute Stroke Care** | Statements are made regarding maintaining high-quality and timely care during these times of reduced or redirected resources.  For hyperacute patients, an emphasis is placed on continuing to provide urgent assessment for thrombolysis and thrombectomy and ongoing management, and to expedite treatment and rehabilitation to avoid delays and minimize length of stay.  Examples of specific strategies include the use of telemedicine, the use of first-line MRI, the use of CTA for all patients with carotid territory symptoms, and the use of artificial Intelligence to speed up image interpretation remotely |
|  | **TIA Triage** | For patients with suspected TIA, the use of telephone triage for all TIA referrals followed by (or at the same time as) virtual TIA clinic, and the rapid prescription of, and delivery of secondary preventative medicines. |
|  | **Outpatients and Rehabilitation** | For outpatients, promoting social distancing and reduced travel to providers while maintaining continuity of care. Non-essential tests can be and canceled or postponed.  Rehabilitation pathways; Rapid discharge from the emergency department, maintain or increase early supported discharge and community rehabilitation programs, and implement telerehabilitation services. |
|  | **Leadership** | A leadership team should be put in place.  Individual departments should consider other specialist pathways that need more support to maintain activity as safely as possible. |
|  | **Principles** | Avoid unnecessary attendances at hospital.  Senior decision-making at the first point of contact should reduce or even prevent the need for further attendances.  Clinicians may need to work in unfamiliar environments or outside their subspecialist areas. They will need to be supported.  Provide simple clear communication within your teams.  Plan ahead for the next stage and consider potential scenarios.  The risk–benefit analysis of everything we do will change and evolve during this epidemic. |
| Yan B  Stroke Society of Australasia statement on Stroke Care during the COVID-19 crisis. April 9, 2020 | **Stroke Care Processes** | COVID-19 pandemic significantly impacts delivery of expert care for stroke patients.  Rapid reperfusion treatment and stroke unit care with rehabilitation support reduce morbidity and mortality.  We must strive to maintain stroke expertise and stroke systems of care during COVID-19 pandemic.  <http://www.strokesociety.com.au/index.php?view=article&catid=41%3Assa&id=505%3Assa-statement-on-stroke-care-during-the-covid-19-&format=pdf&option=com_content> |
| Khosravani H, Rajendram P, Notario L, et al.  *Stroke*;0(0):doi:10.1161/STROKEAHA.120.029838  <https://www.ahajournals.org/doi/pdf/10.1161/STROKEAHA.120.029838> | **Protected Code Stroke** | A protected code stroke algorithm was developed to ensure the safety of healthcare providers during clinical assessment and intervention of patients with hyperacute stroke.  Screening and Prenotification to determine whether to treat as Protected Code Stroke |
|  | **Personal Protection Equipment** | Use of droplet and contact personal protective equipment.  Place a surgical mask on the non-intubated patient.  If patient obtunded, and or requiring high flow O2, consider early intubation and consult with ED/ICU physician for airway management prior to transport to imaging. |
|  | **Crisis Resource Management** | Do not rush into resuscitation room. Slow down when you should.  Designate a safety leader to monitor PPE donning and doffing.  Role designate your team and avoid crowding.  Ensure PPE donned by all team members before starting protected code stroke.  Avoid contamination of other hospital environments en-route to imaging and back. |
| Sharma D, Rasmussen M, Han R, Whalin M, Davis M et al.  Anesthetic Management of Endovascular Treatment of Acute Ischemic Stroke During COVID-19 Pandemic: Consensus Statement from Society for Neuroscience in Anesthesiology & Critical Care (SNACC)  <https://www.snacc.org/wp-content/uploads/2020/04/SNACC-Consensus-Statement-on-Anesthetic-Management-of-Endovascular-Treatment-of-Acute-Ischemic-Stroke-During-COVID-19-Pandemic-with-Image.pdf>  (abbreviated) | **Recommendations for Choice of Anesthetic Technique** | The vast majority of patients will have to be considered “suspected COVID-19” or “unknown COVID-19” when presenting for EVT. Irrespective of the choice of anesthetic technique, we recommend airborne precautions for all these patients.  The choice of anesthetic technique should be carefully individualized accounting for the patient’s neurological and medical status as well as the risk of infection to healthcare personnel.  Not all COVID-19 positive / suspected positive patients require GA for EVT.  In general, the threshold for the use of general anesthesia (GA) for EVT may be reduced during COVID-19 pandemic. If the anesthesiologist has any concerns for possible urgent conversion from monitored anesthesia care (MAC) to GA during EVT, it is advisable to start with GA.  Not all COVID-19 positive / suspected positive patients require GA for EVT. |
|  | **General Anesthetic for EVT** | The following criteria may be used to identify patients who may be preferred candidates for GA during the COVID-19 pandemic:   1. Known / suspected COVID-19 positive patients with AIS who have:    1. acute respiratory distress / hypoxemia / requiring high flow oxygen or    2. active cough or    3. inability to protect airway or    4. active vomiting 2. Posterior circulation / dominant cerebral hemisphere occlusions 3. Severe stroke (NIHSS >15) or GCS <9 4. Agitated / uncooperative patients / aphasic patients   The following criteria may be used to identify patients who may be suitable candidates for MAC during COVID-19 pandemic:   1. Patients who do not have acute respiratory distress or hypoxemia requiring high flow oxygen, are not actively coughing or vomiting, and are able to protect their airway 2. Anterior circulation / non-dominant cerebral hemisphere occlusions 3. NIHSS < 15 and GCS >9 |
|  | **Additional Recommendations** | 5 general recommendations for anesthetic management of EVT in known / suspected COVID-19 positive patients (irrespective of the anesthetic technique) are also provided, addressing special considerations relevant to patients requiring emergent EVT.  14 recommendations for GA / intubation;  7 Recommendations for MAC;  4 recommendations for urgent conversion from MAC to GA. |
|  | **Within Hospital Transport** | Transport for post-EVT imaging should be limited as much as possible.  HEPA filter should remain connected directly to the tracheal tube for intubated patients and capnography used throughout the transport to avoid inadvertent hypo / hyperventilation  Coughing / disconnections of breathing circuits should be avoided as described above  Hemodynamics should be strictly maintained during transport using standard guidelines  Patients who are not ventilated during transport should wear a surgical mask.  Personnel transporting an intubated patient should wear PPE |
| Fraser JF, Arthur A, Chen M, et al.  Society of NeuroInterventional Surgery recommendations for the care of emergent neurointerventional patients in the setting of COVID-19. 2020. (selected)  <http://jsnet.website/contents/200331/SNIS-OVID-Stroke-Protocol.pdf> | **Inclusion for EVT** | The presence of COVID-19 as a public health issue should not alter the inclusion and exclusion criteria for mechanical thrombectomy |
|  | **Intubation of EVT candidates** | **For patients with documented COVID-positive status or Undocumented COVID status**: We recommend standard institutional protocols with a low threshold for intubation of stroke thrombectomy COVID-19 positive patients prior to transport to the angiography suite, ideally in a negative pressure environment. Recommendations related to the use of personal protective equipment (PPE) are provided. |
|  | **Additional Post-Thrombectomy Principles** | All acute ischemic stroke post thrombectomy patients undergo COVID-19 testing if available.  It is recommended that elective and non-urgent cerebrovascular cases be postponed until the pandemic’s peak has been reduced.  For hospitals with multiple angiography suites, one suite should be designated as a “COVID room”  Shift-based allocation of staff and physicians to separate individuals with overlapping skillsets is recommended. |
| Thachil J, Tang N, Gando S et al.  ISTH interim guidance on recognition and management of coagulopathy in COVID-19  doi: 10.1111/JTH.14810  <https://onlinelibrary.wiley.com/doi/epdf/10.1111/jth.14810> March 25, 2020 | **Key statements and guidance**  **D-dimer**  **Low platelets** | The authors acknowledge that guidance statements will be modified with developing knowledge.  Patients who have markedly raised D-dimers (which may be arbitrarily defined as three-fourfold increase), admission to hospital should be considered even in the absence of other severity symptoms since this clearly signify increased thrombin generation.  Thrombocytopenia may also be a prognosticator for mortality, but this has not been a consistent finding. |
|  | **Routine coagulation tests** | Monitoring of the PT, D-dimer, platelet count and fibrinogen can be helpful in determining prognosis in COVID-19 patients requiring hospital admission. |
|  | **VTE prophylaxis** | The only widely available treatment in this respect is prophylactic dose low molecular weight heparin (LMWH) which should be considered in ALL patients (including non-critically ill) who require hospital admission for COVID-19 infection, in the absence of any contraindications (active bleeding and platelet count < 25 x 10^9^ /L; monitoring advised in severe renal impairment; abnormal PT or APTT is not a contraindication). |
|  | **Management of bleeding** | Bleeding is rare in the setting of COVID-19 infection. If bleeding occurs, general ISTH guidance with respect to transfusions may be followed. |
|  | **Experimental therapy** | Other treatments – antithrombin concentrate, recombinant thrombomodulin, hydroxychloroquine) – can only be considered to be experimental at the moment. |
| Zhao, J, Rudd A & Liu R.  Challenges and Potential Solutions of Stroke  Care During the Coronavirus Disease 2019 (COVID-19) Outbreak  *Stroke*; 0: STROKEAHA.120.029701. DOI:10.1161/STROKEAHA.120.029701. | **Organization & Access** | 1. The establishment of stroke networks and care systems able to deliver high-quality emergency stroke care at all times but particularly at times of crisis.  2. The establishment of centralized stroke treatment centers where sufficient stroke care resource can be secured. Although there is a strong case for such centers to be the system of care at all times, it is particularly important at times of medical crisis to have services that can continue to function. |
|  | **Education** | 1. Inform the emergency medical system and the public that these centers will be protected and will remain fully operational even during crises.  2. Improve education of health professionals and the public, especially those who are at high risk of stroke, to recognize stroke and call emergency medical services immediately to be taken to one of the designated stroke centers so as to avoid significant delay in transferring patient from one hospital to the other. |
