# Supplementary material for: Canadian Stroke Best Practice Guidance During the COVID-19 Pandemic
Source: Can J Neurol Sci. 2020 Apr 17:1–5. doi: 10.1017/cjn.2020.74 (PMC7235308; doi:10.1017/cjn.2020.74)
Supplement: Supplementary file 1 [file S0317167120000748sup.zip › S0317167120000748sup002.pdf]

Titre : ***Recommandations canadiennes pour les pratiques optimales de soins de l'AVC*** **pendant la pandémie de COVID-19**

Auteurs

Eric E. Smith, M.D., PH; Anita Mountain, M.D., B.Sc.Erg; Michael D. Hill, M.D., M. Sc.; Theodore H. Wein; M.D.; Dylan Blaquiere, M.D.; Leanne K. Casaubon, M.D., M. Sc.; Elizabeth Linkewich, ergothérapeute agréée (Ontario), M.P.A.; Norine Foley, Dt. P., M. Sc.; Gord Gubitz, M.D.; Anne Simard, B Journ, M. Sc.; Patrice Lindsay, inf. aut., Ph. D., *au nom du comité consultatif canadien sur les pratiques optimales en matière de soins de l'AVC.*

Auteure-ressource

M. Patrice Lindsay, inf. aut., Ph. D.
Directrice, Changement à l'échelle des systèmes et programme sur l'AVC
Éditrice en chef, Recommandations canadiennes pour les pratiques optimales de soins de l'AVC
Fondation des maladies du cœur et de l'AVC du Canada
2300, rue Yonge, bureau 1300, C. P. 2414
Toronto (Ontario) M4P 1E4
Tél. : 647 943-3042
Patrice.Lindsay@heartandstroke.ca

Affiliations

Département de neurosciences cliniques, Cumming School of Medicine, Université de Calgary, Calgary (Alberta) [Smith, Hill]; divisions de médecine physique et de réadaptation, département de médecine, Université Dalhousie (Mountain); département de neurologie et de neurochirurgie, Université McGill, Montréal, (Québec) [Wein]; division de neurologie, département de médecine, Université de Toronto (Casaubon); Programme sur l'AVC de l'hôpital Toronto Western, Réseau universitaire de santé, Toronto (Ontario) [Casaubon]; division de neurologie, Faculté de médecine, Université d'Ottawa, Ottawa (Ontario) [Blacquiere]; WorkHORSE Consulting Inc., London (Ontario) [Foley]; Centre des sciences de la santé Sunnybrook, Centre régional sur l'AVC (Linkewich); Université de Toronto, département de sciences de l'occupation et d'ergothérapie (Linkewich); département de médecine (neurologie), Université Dalhousie (Gubitz); Fondation des maladies du cœur et de l'AVC du Canada (Simard, Lindsay).

Financement

La Fondation des maladies du cœur et de l'AVC a financé l'intégralité de l'élaboration des Recommandations. Aucun financement pour le présent document ne provient d'intérêts commerciaux, y compris des sociétés pharmaceutiques et des fabricants de dispositifs médicaux.

Mots clés : AVC, coronavirus 2019, COVID-19, maladie à coronavirus, lignes directrices sur la pratique clinique, télé-AVC, réadaptation, prévention, application des connaissances.

Recommandations pour les pratiques optimales de soins de l'AVC pendant la pandémie de COVID-19

Recommandations du comité consultatif canadien sur les pratiques optimales en matière de soins de l'AVC de la Fondation des maladies du cœur et de l'AVC du Canada

Introduction

La pandémie mondiale de maladie à coronavirus 2019 (COVID-19) causée par le coronavirus du syndrome respiratoire aigu sévère 2 (SRAS-CoV-2) est apparue comme l'une des plus grandes crises de santé publique du siècle. Les systèmes de santé au Canada sont confrontés à des défis colossaux, tant pour faire face au nombre de patients touchés que pour faire face aux contraintes imposées par les mesures de confinement telles que la distanciation sociale, la quarantaine et la protection personnelle. Les soins de l'AVC à l'échelle tant nationale que mondiale évoluent rapidement pour relever ces défis[1-7] (voir l'annexe A).

Ce document fournit des lignes directrices sur la mise en œuvre de soins de l'AVC basés sur des données probantes pendant la pandémie de la COVID-19, en se fondant sur l'avis des experts du comité consultatif canadien sur les pratiques optimales en matière de soins de l'AVC. Malgré des données incomplètes qui évoluent rapidement, nous offrons des lignes directrices précoces sans toutefois fournir des recommandations formelles ni des niveaux de preuves, car des changements urgents sont nécessaires. Ces lignes directrices sont fondées sur l'avis d'experts et les premières expériences communes en matière de réorganisation des systèmes de soins de l'AVC au moment de la rédaction du présent document (13 avril 2020).

Ce document est guidé par deux grands principes. Premièrement, l'AVC reste une urgence médicale et doit être traité comme tel. Deuxièmement, les soins de l'AVC sont très efficaces. Les recommandations pour les pratiques optimales de soins de l'AVC restent tout autant fondées sur des données probantes et pertinentes, même si la logistique et les flux de travail doivent changer pour s'adapter à la situation pandémique. Les soins de l'AVC fondés sur des données probantes réduisent la mortalité et la durée de l'hospitalisation, améliorent les résultats fonctionnels et préviennent les récidives, ce qui soulage le système de santé[8-10]. Les chefs de file en matière d'AVC sont tous d'accord pour dire que ces soins ne doivent pas être intentionnellement arrêtés ou suspendus (annexe 1).

Partout dans le monde, les centres de soins de l'AVC rapportent une diminution du nombre de personnes qui ont des symptômes de l'AVC et qui se présentent aux services des urgences pour recevoir des soins, particulièrement des cas d'accident ischémique transitoire (AIT) ou de symptômes bénins. Les causes de cette situation ne sont pas claires à l'heure actuelle. Il existe

des preuves anecdotiques que les tendances de participation des patients aux services de santé pourraient changer pendant la pandémie. De telles diminutions peuvent susciter de nouvelles préoccupations puisque les personnes réticentes à obtenir des soins médicaux peuvent être à risque de récidive avec des conséquences plus sérieuses et durables sur les plans physique, cognitif et émotionnel sans une évaluation et un traitement rapides.

Sensibilisation, reconnaissance et réponse à l'AVC

L'AVC est une urgence médicale. Cela ne change pas malgré la pandémie de la COVID-19. Les campagnes de sensibilisation du public et les processus en place pour l'intervention d'urgence du système médical en matière d'AVC doivent être maintenus. Des efforts actifs de sensibilisation du public sont nécessaires afin de renforcer ce message et de réduire les délais avant la demande d'assistance médicale.

Soins de l'AVC en phase hyperaiguë

L'AVC en phase aiguë présente un risque puisque l'équipe de prise en charge de l'AVC doit être en contact étroit avec les patients, dont un grand nombre n'aura pas de statut connu concernant la COVID-19. Les patients qui ne sont pas en mesure de répondre aux questions de dépistage de la COVID-19 à cause d'aphasie, de problèmes cognitifs ou d'encéphalopathie doivent être traités comme des cas positifs suspectés. Un équipement de protection individuelle (EPI) doit être porté, conformément aux politiques locales. Des lignes directrices pour une séquence de code d'AVC protégé (« Protected Code Stroke pathway ») ont été proposées, mettant l'accent sur le dépistage et l'utilisation d'EPI approprié, où chaque membre de l'équipe comprend son rôle précis afin de minimiser le risque potentiel d'exposition à la COVID-19[1,4]. La télémédecine peut être utilisée pour la consultation en cas d'AVC en phase aiguë afin d'éviter d'exposer les membres de l'équipe et de réduire l'utilisation des EPI.

La thrombectomie endovasculaire (TE) est un traitement très efficace de l'AVC. Elle est indiquée pour les patients d'AVC ischémique grave et à risque d'instabilité respiratoire, de vomissements, d'aspiration et de toux qui pourraient augmenter la propagation de gouttelettes virulentes. Pour éviter le risque d'intubation d'urgence dans la salle d'angiographie de TE, avec un potentiel de propagation virale, il peut être approprié de prendre des décisions assez tôt concernant le besoin d'intubation. Si nécessaire, celle-ci doit être effectuée de façon contrôlée et optionnelle dans une salle à pression négative, avant le transfert dans la salle d'angiographie. Cela n'implique pas de devoir intuber plus de patients et nous continuons à recommander l'anesthésie surveillée sauf en cas d'indication clinique pour une intubation[5]. De même, de récentes lignes directrices de plusieurs sociétés indiquent que l'intubation n'est pas nécessaire dans tous les cas suspectés ou confirmés de COVID-19[11,12]. Une sédation minimale doit être

utilisée pour les patients suspectés d'avoir la COVID-19, afin de limiter les ventilations par ballon-masque, une procédure génératrice d'aérosols[11].

La demande en lits en unité de soins intensifs (USI) pourrait dépasser le nombre de lits disponibles pendant l'afflux de patients atteints de la COVID-19. Il convient de considérer la mortalité prévue liée à l'AVC comme un critère d'admission en USI. Toutefois, de nombreux patients ayant subi un AVC, y compris un AVC hémorragique, peuvent être sauvés grâce aux soins intensifs et auront un taux de mortalité prévu inférieur à celui des patients du même âge atteints d'un syndrome de détresse respiratoire aiguë à cause de la COVID-19. Comme le recommandent les éthiciens, les décisions relatives au triage devraient être fondées sur des données objectives concernant le risque de mortalité sans présumer la qualité de vie des survivants d'AVC[13]. Le triage pour l'admission en USI ne doit être effectué qu'après avoir essayé les procédures d'urgence pour remédier à l'état du patient, y compris la thrombolyse intraveineuse et la TE pour les cas d'AVC ischémique en phase aiguë et le traitement de l'hydrocéphalie pour l'AVC hémorragique.

Nous encourageons les cliniciens à modifier leurs pratiques, sans toutefois s'éloigner des soins fondés sur des données probantes[5]. Nous ne recommandons pas de modifier les protocoles d'angiographie par tomodensitométrie pour l'AVC en phase aiguë en incluant une tomodensitométrie de la poitrine pour rechercher des signes de la COVID-19. La valeur de la tomodensitométrie de la poitrine pour le diagnostic de la COVID-19, prenant en compte les taux de faux positifs et de faux négatifs, n'est pas concluante pour le moment. Nous ne recommandons pas non plus de substituer le ténectéplase à l'altéplase pour la thrombolyse bien qu'il soit plus pratique à infuser et demande ainsi de passer moins de temps au contact du patient. Les données affirmant que le ténectéplase est équivalent à l'altéplase sont insuffisantes et la dose optimale n'est pas claire.

Messages clés

1. L'AVC est une urgence médicale, même en temps de pandémie; les lignes directrices pour l'AVC fondées sur des données probantes doivent toujours être suivies.
2. Il est nécessaire de continuer à sensibiliser les gens au fait que l'AVC est une urgence médicale et que les personnes doivent obtenir une assistance médicale immédiate, malgré les préoccupations entourant la COVID-19.
3. Les équipes d'intervention à l'AVC en phase hyperaiguë restent disponibles pour traiter les AVC en phase aiguë.
4. Des changements dans les processus de flux de travail sont nécessaires dans le cadre d'un modèle de code d'AVC protégé (« Protected Code Stroke model »).
5. L'intubation n'est pas nécessaire pour tous les cas suspectés ou confirmés de COVID-19 traités par TE.

Soins hospitaliers et unité en soins de l'AVC

Les unités en soins de l'AVC, définis comme des soins par une équipe interdisciplinaire expérimentée avec un regroupement de patients dans une unité d'hospitalisation spécifique, réduisent les incapacités et sauvent des vies. Les difficultés pour fournir des soins en unité d'AVC pendant la pandémie comprennent la diminution de personnel en raison de la maladie ou de réaffectations, et l'admission potentielle de patients d'AVC atteints de la COVID-19 dans des services hospitaliers généraux au lieu d'unités en soins de l'AVC.

Les ressources des hôpitaux en matière de soins de santé sont déjà de plus en plus sollicitées, notamment avec la réaffectation de professionnels de la santé hautement qualifiés. Par conséquent, il est possible que des experts non spécialisés en soins de l'AVC soient responsables des unités d'AVC. Les hôpitaux devraient donc développer des approches solides, basées sur le travail d'équipe, afin d'optimiser les pratiques de soins pour les patients d'AVC en utilisant les capacités de tous les membres de l'équipe. Les patients ayant reçu un double diagnostic d'AVC en phase aiguë et de COVID-19 peuvent être admis dans une unité non spécialisée en soins de l'AVC avec des professionnels de la santé moins expérimentés dans ces soins et la réadaptation précoce[5]. Dans ces cas, des processus devraient être mis en place pour la consultation d'experts sur l'AVC et pour la formation sur les pratiques optimales en matière de soins de l'AVC[14]. La formation est particulièrement importante pour la reconnaissance, l'évaluation et la gestion de la dysphagie, de l'aphasie, des déficits cognitifs, de la manipulation et du positionnement des extrémités hémiplégiques, de la prévention de la thromboembolie veineuse, des transferts et de la prévention des chutes. Tout le personnel interdisciplinaire devrait également recevoir une formation de base sur l'évaluation des signes et symptômes de l'AVC dans le cadre de la surveillance d'une éventuelle transformation de l'AVC. Elle devrait inclure les outils de dépistage comme VITE (Visage, Incapacité, Trouble de la parole, Extrême urgence)[15] et les protocoles pour les mesures à prendre à l'hôpital en cas d'identification de signes et symptômes d'AVC. Une formation et un soutien accentués peuvent être nécessaires pour les professionnels de la santé qui doivent s'occuper de patients ayant subi une hémorragie intracérébrale ou une hémorragie sous-arachnoïdienne.

Idéalement, une fois le traitement de reperfusion en phase hyperaiguë (thrombolyse et/ou TE) administré, les soins doivent être prodigués dans une unité de surveillance intensive ou de soins intensifs. Si l'accès aux lits de soins intensifs est limité, ces soins peuvent être prodigués dans une unité de soins où le soutien adéquat est disponible. D'une manière générale, cela inclut une surveillance accrue des patients, en particulier dans les 24 heures suivant un traitement en phase aiguë; une formation de l'équipe interdisciplinaire sur les soins aux patients ayant subi une thrombolyse et une TE; et une communication claire entre les membres de l'équipe lorsqu'il est question de l'état clinique des patients. Ces derniers doivent être soignés dans un endroit bien visible depuis le hall et, idéalement, faire l'objet d'une télésurveillance cardiaque.

Messages clés
1. Les patients ayant subi un AVC devraient continuer à être soignés dans des unités spécialisées en soins de l'AVC aigu, si possible.
2. Il peut s'avérer nécessaire que les experts non spécialisés dans les soins aux patients d'AVC reçoivent une formation professionnelle de base afin de garantir la sécurité des patients et d'optimiser le rétablissement de ces derniers.
3. Si l'accès aux lits en soins intensifs est limité, ces soins peuvent être prodigués dans une unité d'AVC où le soutien adéquat est disponible.

Réadaptation après l'AVC

L'accès aux soins de réadaptation a été considérablement réduit pendant la pandémie de la COVID-19[16,17]. Les personnes ayant subi un AVC renvoyées directement dans la communauté après avoir reçu des soins de courte durée pourraient avoir un accès limité à une réadaptation après l'AVC. Le séjour des personnes qui suivent une réadaptation post-AVC en milieu hospitalier pourrait être écourté[1,18]. Il reste essentiel que les personnes ayant subi un AVC aient encore accès aux congés précoces assistés et à des soins spécialisés en milieu hospitalier, externe ou communautaire. La réadaptation post-AVC est essentielle pour atteindre un niveau fonctionnel optimal sur les plans physique, cognitif, émotionnel, communicatif et social après l'AVC, ainsi que pour prévenir ou ralentir une éventuelle dégradation fonctionnelle et des problèmes de santé secondaires[19].

Les équipes responsables de la réadaptation doivent continuer à offrir des soins fondés sur des données probantes aux patients[19]. Afin de garantir un accès sécuritaire aux soins, ces équipes doivent savoir comment utiliser correctement les EPI et respecter rigoureusement les procédures de lutte contre les infections lors des traitements qui requièrent un contact direct, des équipements partagés ou des espaces communs[17]. Les composantes essentielles des soins de réadaptation devraient être conformes aux recommandations de santé publique sur la distanciation sociale, en tenant compte d'outils comme les vidéoconférences.

La téléréadaptation est une façon efficace et reconnue d'offrir des services de réadaptation en milieux externe et communautaire, et elle revêt une importance particulière pendant la pandémie de la COVID-19[20-22]. Lors de la planification du congé, la téléréadaptation devrait être envisagée pour les rencontres familiales, la formation des habiletés et l'éducation des familles et des aidants, l'évaluation du milieu de vie, la surveillance des patients et les traitements en consultation externe. Si la téléréadaptation est prévue pour un traitement en consultation externe, l'éducation, la formation des habiletés et la mise en place de la plate-forme de téléréadaptation choisie pour les patients, les familles et les aidants doivent être effectuées avant le congé. Il faut également fournir aux patients des recommandations claires concernant leur congé[19] et des instructions pour leur permettre de poursuivre leur réadaptation à domicile.

Il convient d'envisager un suivi plus précoce des patients dont la durée de séjour est raccourcie par les politiques liées à la COVID-19, afin de détecter plus tôt les complications potentielles ou une possible dégradation fonctionnelle. La télémédecine peut également aider à déterminer les patients dont l'évolution des besoins et de l'état de santé nécessite une évaluation en personne[23].

Les personnes résidant dans la communauté et ayant des AVC chroniques auront encore besoin d'accéder aux services de réadaptation. Les professionnels en réadaptation doivent s'assurer qu'il existe des processus de triage et qu'ils peuvent répondre aux besoins des patients pour prévenir la dégradation fonctionnelle et les complications pendant la pandémie de la COVID-19. De même, les injections de toxine botulique à domicile devraient être envisagées seulement si l'EPI approprié est accessible, si le patient risque de ressentir une douleur et de présenter une dégradation fonctionnelle, ou si les injections augmentent le fardeau des aidants.

Messages clés

1. Il est essentiel que les personnes ayant subi un AVC aient toujours accès aux congés précoces assistés et à des soins spécialisés en milieu hospitalier, externe ou communautaire.
2. Les composantes essentielles des soins de réadaptation devraient être conformes aux recommandations de santé publique sur la distanciation sociale de manière à protéger le personnel et les patients lorsque des contacts directs sont nécessaires.
3. La téléréadaptation est une façon efficace et reconnue d'offrir des services de réadaptation en milieux externe et communautaire, et elle revêt une importance particulière pendant la pandémie de la COVID-19.

Soins de prévention secondaire de l'AVC

En temps de pandémie, l'accès aux services spécialisés de prévention secondaire de l'AVC[24] peut être limité. De nombreuses autorités sanitaires ont fortement déconseillé l'évaluation en personne des patients suivis en externe. Aussi, la plupart des soins de prévention de l'AVC devront être offerts par l'intermédiaire de la télémédecine, et les évaluations devront être modelées en fonction des concepts définis dans la liste de contrôle post-AVC et des éléments essentiels des soins de prévention[25,26]. Les défis de prévention secondaire liés à la gestion du mode de vie, comme l'alimentation, l'exercice, le poids, la consommation d'alcool et le tabagisme, doivent être abordés et peuvent être affectés par les recommandations des autorités sanitaires, qui nous disent de rester à la maison. Les patients, les familles et les aidants doivent être sensibilisés et connaître les stratégies et les ressources relatives à l'autogestion de la maladie[27]. Il est possible d'effectuer certaines étapes d'un examen neurologique par l'intermédiaire de la télémédecine grâce à une évaluation directe (p. ex., état

mental et élocution), à une observation (p. ex., évaluation d'une partie des nerfs crâniens, de la fonction motrice des membres, de la coordination et de la démarche) ou à une personne qui accompagne le patient (examen sensitif)[28]. Cet examen est essentiel pour évaluer la possibilité d'une récidive d'AVC. Il convient de demander aux patients s'ils ont accès à une unité de pression artérielle à domicile et à un glucomètre (le cas échéant) pour effectuer la surveillance continue des objectifs de prévention secondaire.

Pour les cas nécessitant des soins en personne, les cliniques d'évaluation rapide de l'AIT ou de prévention de l'AVC devront mettre en place des mécanismes permettant de vérifier si les patients présentent des symptômes de la COVID-19 avant leur arrivée. De plus, les mesures de protection et les EPI appropriés devront y être utilisés. Les personnes qui se présentent dans les 24 heures doivent subir d'urgence tous les examens nécessaires, y compris une tomodensitométrie, une angiographie par tomodensitométrie et une électrocardiographie. Il est conseillé aux professionnels de la santé qui demandent une imagerie neurovasculaire d'urgence de communiquer directement avec un radiologue afin de s'assurer que l'examen d'imagerie peut être réalisé rapidement, car les flux normaux de demandes risquent d'être interrompus. D'autres examens doivent être effectués dès que possible conformément aux lignes directrices et en tenant compte du fait que certains services de diagnostic (p. ex., échocardiographie) pourraient ne pas être disponibles pendant la pandémie. Les personnes qui se présentent après le délai de 24 heures doivent subir des examens tels que définis dans l'algorithme des pratiques optimales de soins de l'AVC[24]. On suggère d'admettre directement les patients nécessitant une hospitalisation, afin de réduire la charge des urgences.

Messages clés

1. Les services de prévention secondaire et de suivi doivent continuer à être mis en œuvre pour réduire l'incidence des AVC récurrents, et les flux de demandes doivent être revus.
2. Les examens effectués grâce à la télémédecine doivent être modelés en fonction des concepts définis dans la liste de contrôle post-AVC et des éléments essentiels des soins de prévention (se reporter aux pages Web sur les Recommandations).
3. Les personnes qui se présentent dans les 24 heures doivent subir d'urgence tous les examens nécessaires, y compris une tomodensitométrie, une angiographie par tomodensitométrie et une électrocardiographie.

Télé-AVC dans le continuum de soins

Devant la situation pandémique, la télémédecine a été rapidement adoptée par de nombreux systèmes de santé pour faciliter la prestation de soins tout en respectant la distanciation sociale et en réduisant le risque de transmission nosocomiale de la maladie. Les systèmes de télé-AVC pour les soins de l'AVC en phase hyperaiguë et l'aide à la prise de décision relative aux soins de

thrombolyse et de TE sont bien établis[20]. La télémédecine peut fournir un accès à distance aux spécialistes de l'AVC, ce qui permet d'éviter les transferts vers des centres de soins tertiaires. Elle permet de réduire le nombre d'EPI utilisé et préserve la main-d'œuvre spécialisée en évitant tout risque d'exposition et d'infection[1]. Dans les établissements ne disposant pas des systèmes actuels de télé-AVC, d'autres outils de télémédecine comme les logiciels de vidéoconférence, le partage d'imagerie et les consultations téléphoniques peuvent être mis en place pour aider à la prise de décision en cas d'AVC en phase hyperaiguë[29,30].

Des trousses d'outils basées sur les données probantes actuelles et l'opinion d'experts sont offertes dans les Recommandations pour guider les services qui doivent se tourner rapidement vers les soins virtuels[20,31].

Les dispensateurs de soins de l'AVC et les systèmes de santé doivent être conscients des obstacles potentiels à l'accès aux soins. Par exemple, un patient ou un aidant pourrait ne pas avoir accès à un appareil ou à un service Internet fiable. Il faut également prendre en compte les obstacles à l'utilisation de la technologie chez les personnes ayant subi un AVC et présentant des déficits cognitifs ou physiques ou des difficultés communicationnelles. D'autres déterminants sociaux de la santé, tels que l'accès à un logement stable, peuvent également poser des défis relatifs à l'utilisation de modalités virtuelles pour recevoir des services de santé. Des rencontres téléphoniques ou la participation des membres de la famille pendant le processus d'évaluation pourraient permettre de surmonter ces obstacles.

Messages clés

1. Les systèmes de télé-AVC pour les soins de l'AVC en phase hyperaiguë et l'aide à la prise de décision relative aux soins de thrombolyse et de TE sont bien établis et devraient être accessibles dans toutes les régions.
2. Des trousses d'outils basées sur les données probantes actuelles et l'opinion d'experts sont offertes dans les Recommandations pour guider les services qui doivent se tourner rapidement vers les soins virtuels.
3. Les obstacles à l'accès aux soins doivent être pris en compte et des solutions de contournement doivent être appliquées.

Résumé

La pandémie de la COVID-19 a radicalement changé les processus de soins de l'AVC et d'accès aux patients. La nature et la qualité des soins de l'AVC apportés aux personnes ayant subi un AVC dans l'ensemble du continuum ont un effet éprouvé sur les résultats à long terme. C'est pourquoi les normes et l'exhaustivité des soins à ces personnes doivent être préservées, faute de quoi le taux d'AVC récurrents et le nombre de déficits fonctionnels, cognitifs et sociaux permanents augmenteront et ajouteront un fardeau additionnel sur un système déjà surchargé.

Dans l'ensemble, les mesures visant à maintenir les pratiques optimales de soins de l'AVC devraient être mises en œuvre dans le cadre de la planification de la pandémie, et ce, dans l'ensemble du système de santé. De nouveaux modèles de soins permettent d'offrir aux personnes qui en ont besoin un accès continu aux soins de l'AVC pendant et après la pandémie de la COVID-19.

Disclosures

EES, MPL, DB, THW, AS, NF, EL have no conflicts of interest to disclose.

AM reports involvement in clinical trial grants from Brain Canada, Heart and Stroke Foundation of Canada, and the Canadian Partnership for Stroke Recovery; MDH reports a relationship with NoNO Inc as an unpaid advisor, Boehringer-Ingelheim as a paid advisor, and holds research grants from CIHR, Alberta Innovates, Heart and Stroke Foundation of Canada, Heart and Stroke Foundation of Alberta, multiple industry partners for clinical trials managed through the University of Calgary; LKC participated in an advisory board for Bayer in 2018, and is an independent neurological assessor for patients in SURTAVI trial; GG is a collaborator in the ESCAPE-NA1 trial with the University of Calgary.

Statement of Authorship:

EES is first author, led the hyperacute stroke management section and contributed to all aspects of the concept, design, writing and editing of the manuscript; AM led the rehabilitation section and contributed to all aspects of the concept, design, writing and editing of the manuscript; MDH contributed to the hyperacute stroke management section and contributed to all aspects of the concept, design, writing and editing of the manuscript; THW led the prevention section and contributed to all aspects of the concept, design, writing and editing of the manuscript; DB led the telemedicine section and contributed to all aspects of the concept, design, writing and editing of the manuscript and supports digital knowledge translation resources; LKC led the inpatient stroke care section and contributed to all aspects of the concept, design, writing and editing of the manuscript; EL contributed to the inpatient stroke care section and contributed to aspects of the concept, design, writing and editing of the manuscript; NF conducted the systematic evidence reviews, and contributed to the editing and preparation of the manuscript; GG contributed to aspects of the writing and editing of the manuscript, and supports digital knowledge translation resources; AS contributed to all aspects of the concept, design and editing of the manuscript and supports digital knowledge translation resources; MPL is senior and corresponding author, coordinated and contributed to all aspects of the concept, design, writing and editing of the manuscript, and digital knowledge translation resources.

Acknowledgements

Heart & Stroke gratefully acknowledges the Canadian Stroke Best Practices Advisory Council members, including Eric Smith, Anita Mountain, Leanne K Casaubon, Gord Gubitz, Dar Dowlatshahi, Dylan Blacquiere, Thalia Field, Louise Clement, Farrell Leibovitch, Christine Papoushek, Jeffrey Habert, Barbara Campbell, Joyce Fung, Michael Hill, Tim Hillier, Thomas Jeerakathil, Eddy Lang, Pascale Lavoie, Beth Linkewich, Colleen O'Connell, Melanie Penn, Jai Jai Shankar, Debbie Timpson, Theodore Wein, and Katie White for their guidance throughout this process. We acknowledge and thank Norine Foley and the evidence analysis team at workHORSE; Laurie Charest of Heart & Stroke for her coordination of the CSBPR teams and processes; and the Heart & Stroke internal teams who contributed to the publication and dissemination of these recommendations including Communications, Translation, Promote Recovery, Creative Services, and Digital Solutions.

Références

1. Temporary Emergency Guidance to US Stroke Centers During the COVID-19 Pandemic. Stroke. 2020. DOI: 10.1161/strokeaha.120.030023

2. Macchine D. Clinical guide for the management of stroke patients during the coronavirus pandemic 23 March 2020; Version 1. 2020. https://www.england.nhs.uk/coronavirus/wp-content/uploads/sites/52/2020/04/C0033-_Specialty-guide_-Stroke-and-coronavirus-V1-update_16-April-003.pdf (Accessed April 16, 2020).

3. Yan B. Stroke Society of Australasia statement on Stroke Care during the COVID-19 crisis.; 2020. https://www.strokesociety.com.au/index.php?option=com_content&view=article&id=505%3Assa-statement-on-stroke-care-during-the-covid-19-&catid=41%3Assa&Itemid=177 (Accessed April 9, 2020).

4. Khosravani H, Rajendram P, Notario L, Chapman MG, Menon BK. Protected Code Stroke: Hyperacute Stroke Management During the Coronavirus Disease 2019 (COVID-19) Pandemic. *Stroke.* 2020:Strokeaha120029838.

5. Sharma D, Rasmussen M, Han R, et al. Anesthetic Management of Endovascular Treatment of Acute Ischemic Stroke During COVID-19 Pandemic: Consensus Statement from Society for Neuroscience in Anesthesiology & Critical Care (SNACC). 2020. https://www.snacc.org/wp-content/uploads/2020/04/SNACC-Consensus-Statement-on-Anesthetic-Management-of-Endovascular-Treatment-of-Acute-Ischemic-Stroke-During-COVID-19-Pandemic-with-Image.pdf (asscessed April 9, 2020).

6. Fraser JF, Arthur A, Chen M, et al. Society of NeuroInterventional Surgery recommendations for the care of emergent neurointerventional patients in the setting of COVID-19. 2020. https://www.snisonline.org/wp-content/uploads/2020/03/SNIS-COVID-Stroke-Protocol.pdf (Accessed April 9, 2020).

7. Thachil J, Tang N, Gando S, et al. ISTH interim guidance on recognition and management of coagulopathy in COVID-19. Journal of Thrombosis and Haemostasis. 2020. DOI:10.1111/JTH.14810.

8. Schwamm LH, Chumbler N, Brown E, et al. Recommendations for the Implementation of Telehealth in Cardiovascular and Stroke Care: A Policy Statement From the American Heart Association. *Circulation.* 2017;135(7):e24-e44.

9. Lindsay P, Furie KL, Davis SM, Donnan GA, Norrving B. World Stroke Organization global stroke services guidelines and action plan. *Int J Stroke.* 2014;9 Suppl A100:4-13.

10. Zhao J, Rudd A, Liu R. Challenges and Potential Solutions of Stroke Care During the Coronavirus Disease 2019 (COVID-19) Outbreak. *Stroke.*0(0):STROKEAHA.120.029701.

11. Fraser JF, Arthur A, Chen M, et al. Society of NeuroInterventional Surgery recommendations for the care of emergent neurointerventional patients in the setting of COVID-19. Society of NeuroInterventional Surgery. http://jsnet.website/contents/200331/SNIS-COVID-Stroke-Protocol.pdf. Published 2020. Accessed April 9, 2020.

12. Boulanger JM, Lindsay MP, Gubitz G, et al. Canadian Stroke Best Practice Recommendations for Acute Stroke Management: Prehospital, Emergency Department, and Acute Inpatient Stroke Care, 6th Edition, Update 2018. *Int J Stroke.* 2018;13(9):949-984.

13. Emanuel EJ, Persad G, Upshur R, et al. Fair Allocation of Scarce Medical Resources in the Time of Covid-19. N Engl J Med. 2020. DOI: 10.1056/NEJMsb2005114.

14. Pratiques optimales de soins de l'AVC. « Agir en vue de soins optimaux communautaires et de longue durée de l'AVC : une ressource pour les dispensateurs de soins de santé », Fondation des maladies du cœur et de l'AVC. Publié en novembre 2015. Disponible : https://www.heartandstroke.ca/-/media/pdf-files/what-we-do/publications/001-16-hsf_f15_tacls_booklet_fr_v17_linked.ashx?rev=9e8d6c38b7424d4d9117f752eb7677a6.

15. American Stroke Association. « Use the letters in "F.A.S.T." to spot stroke signs and know when to call 9-1-1 ». (Consulté le 12 avril 2020). Disponible https://www.stroke.org/en/about-stroke/stroke-symptoms.

16. Boldrini P, Kiekens C, Bargellesi S, et al. First impact on services and their preparation. "Instant paper from the field" on rehabilitation answers to the Covid-19 emergency. Eur J Phys Rehabil Med. 2020. DOI: 10.23736/S1973-9087.20.06303-0.

17. McNeary L, Maltser S, Verduzco-Gutierrez M. Navigating Coronavirus Disease 2019 (Covid-19) in Physiatry: A CAN report for Inpatient Rehabilitation Facilities. Pm r. 2020. DOI: 10.1002/pmrj.12369.

18. Negrini S, Ferriero G, Kiekens C, Boldrini P. Facing in real time the challenges of the Covid-19 epidemic for rehabilitation. Eur J Phys Rehabil Med. 2020. DOI: 10.23736/S1973-9087.20.06286-3.

19. Teasell R, Salbach NM, Foley N, et al. Canadian Stroke Best Practice Recommendations: Rehabilitation, Recovery, and Community Participation following Stroke. Part One: Rehabilitation and Recovery Following Stroke; 6th Edition Update 2019. *Int J Stroke.* 2020:1747493019897843.

20. Blacquiere D, Lindsay MP, Foley N, et al. Canadian Stroke Best Practice Recommendations: Telestroke Best Practice Guidelines Update 2017. *Int J Stroke.* 2017;12(8):886-895.

21. Caughlin S, Mehta S, Corriveau H, et al. Implementing Telerehabilitation After Stroke: Lessons Learned from Canadian Trials. Telemed J E Health. 2019. DOI: 10.1089/tmj.2019.0097.

22. Laver KE, Adey-Wakeling Z, Crotty M, Lannin NA, George S, Sherrington C. Telerehabilitation services for stroke. *Cochrane Database Syst Rev.* 2020;1:Cd010255.

23. Portnoy J, Waller M, Elliott T. Telemedicine in the Era of COVID-19. J Allergy Clin Immunol Pract. 2020. DOI: 10.1016/j.jaip.2020.03.008.

24. Wein T, Lindsay MP, Cote R, et al. Canadian stroke best practice recommendations: Secondary prevention of stroke, sixth edition practice guidelines, update 2017. *Int J Stroke.* 2018;13(4):420-443.

24. Wein T, Lindsay MP, Cote R, et al. Canadian stroke best practice recommendations: Secondary prevention of stroke, sixth edition practice guidelines, update 2017. *Int J Stroke.* 2018;13(4):420-443.

25. Pratiques optimales de soins de l'AVC au Canada. *Éléments fondamentaux des services de prévention de l’AVC*, « Prévention secondaire de l'AVC », Fondation des maladies du cœur et de l'AVC. Publié en 2017. Disponible (en anglais) : https://www.heartandstroke.ca/-/media/1-stroke-best-practices/resources/professional-resouces/hsf-csbpr-core-elements-of-delivery-of-stroke-prevention-services.ashx?rev=fcff9f51ac364646a5d4fda1f02b7fa8.

26. Pratiques optimales de soins de l'AVC au Canada. « Liste de contrôle post-AVC ». Conçu par le Global Stroke Community Advisory Panel (2012), endossé par l’Organisation mondiale de l’AVC, adapté par l’équipe de rédaction des *Recommandations canadiennes pour les pratiques optimales de soins de l’AVC* de la Fondation des maladies du cœur et de l’AVC (2014). Disponible : https://www.heartandstroke.ca/-/media/1-stroke-best-practices/resources/french-patient-resources/hsfpoststrokechecklistfrenchmay42.ashx?rev=432e668697174ca789fccb9ae0c0e35a.

27. Pratiques optimales de soins de l'AVC au Canada. « Ressources pour les patients et les aidants », Fondation des maladies du cœur et de l'AVC. (Consulté le 13 avril 2020). Disponible : https://www.pratiquesoptimalesavc.ca/ressources/ressources-pour-les-patients-et-les-aidants-naturels.

28. Klein BC, Busis NA. COVID-19 is catalyzing the adoption of teleneurology. Neurology. 2020. DOI: 10.1212/WNL.0000000000009494.

29. Meyer BC, Raman R, Hemmen T, et al. Efficacy of site-independent telemedicine in the STRokE DOC trial: a randomised, blinded, prospective study. *Lancet Neurol.* 2008;7(9):787-795.

30. Powers WJ, Rabinstein AA, Ackerson T, et al. 2018 Guidelines for the Early Management of Patients With Acute Ischemic Stroke: A Guideline for Healthcare Professionals From the American Heart Association/American Stroke Association. *Stroke.* 2018;49(3):e46-e110

31. Pratiques optimales de soins de l'AVC au Canada. « Trousse d'outils de mise en œuvre de la télé-AVC », Fondation des maladies du cœur et de l'AVC. Mis à jour en 2017 (consulté le 13 avril 2020). Disponible (en anglais) : https://www.heartandstroke.ca/-/media/1-stroke-best-practices/resources/professional-resouces/csbpr2017_telestroketoolkit-updated.ashx?rev=72b13c7c8c8a45a2be07a6758146756b.
